# Benchmark of Intraoperative Activity in Cardiac Surgery: A Comparison between Pre- and Post-Operative Prognostic Models

**DOI:** 10.3390/jcm11113231

**Published:** 2022-06-06

**Authors:** Anna Zamperoni, Greta Carrara, Massimiliano Greco, Carlotta Rossi, Elena Garbero, Giovanni Nattino, Giuseppe Minniti, Paolo Del Sarto, Guido Bertolini, Stefano Finazzi, Cardiac Surgical Intensive Care Writing Committee (GiViTI)

**Affiliations:** 1 Cà Foncello Hospital, AULSS2 Treviso, 31100 Treviso, Italy; anna.zamperoni@aulss2.veneto.it (A.Z.); giuseppe.minniti@aulss2.veneto.it (G.M.); 2 Istituto di Ricerche Farmacologiche Mario Negri IRCCS, 20156 Milan, Italy; greta.carrara@marionegri.it (G.C.); carlotta.rossi@marionegri.it (C.R.); elena.garbero@marionegri.it (E.G.); giovanni.nattino@marionegri.it (G.N.); guido.bertolini@marionegri.it (G.B.); stefano.finazzi@marionegri.it (S.F.); 3 Department of Biomedical Sciences, Humanitas University, 20090 Milan, Italy; 4 Department of Anesthesiology and Intensive Care, IRCCS Humanitas Research Hospital, 20089 Milan, Italy; 5 Department of Critical Care, G. Pasquinucci Heart Hospital, Fondazione Toscana G. Monasterio, 54100 Massa, Italy; paolo.delsarto@ftgm.it

**Keywords:** cardiac surgery, mortality, anesthesia, forecasting, heart valve disease, bypass surgery, coronary artery

## Abstract

Objectives: Despite its large diffusion and improvements in safety, the risks of complications after cardiac surgery remain high. Published predictive perioperative scores (EUROSCORE, STS, ACEF) assess risk on preoperative data only, not accounting for the intraopertive period. We propose a double-fold model, including data collected before surgery and data collected at the end of surgery, to evaluate patient risk evolution over time and assess the direct contribution of surgery. Methods: A total of 15,882 cardiac surgery patients from a Margherita-Prosafe cohort study were included in the analysis. Probability of death was estimated using two logistic regression models (preoperative data only vs. post-operative data, also including information at discharge from the operatory theatre), testing calibration and discrimination of each model. Results: Pre-operative and post-operative models were built and demonstrate good discrimination and calibration with AUC = 0.81 and 0.87, respectively. Relative difference in pre- and post-operative mortality in separate centers ranged from −0.36 (95% CI: −0.44–−0.28) to 0.58 (95% CI: 0.46–0.71). The usefulness of this two-fold preoperative model to benchmark medical care in single hospital is exemplified in four cases. Conclusions: Predicted post-operative mortality differs from predicted pre-operative mortality, and the distance between the two models represent the impact of surgery on patient outcomes. A double-fold model can assess the impact of the intra-operative team and the evolution of patient risk over time, and benchmark different hospitals on patients subgroups to promote an improvement in medical care in each center.

## 1. Introduction

Due to the ageing of the population, cardiovascular diseases represent a larger proportion of the total health care expenditure. Quality of life and working ability of an individual with chronic cardiac conditions can significantly improve after cardiac surgery [1,2]. This leads to improved patient health, while also reducing social and medical costs in long-term cardiac disease.

Despite its advantages, cardiac surgery is a high-complexity and high-cost surgery [3]. Accordingly, the availability of a predictive tool to reduce potential complications would be useful not only to weigh pre-operative risk, but also to titrate the evolution of the perioperative risk during the early phases of postoperative recovery.

The perioperative risk scores largely in use today are developed considering pre-operatory variables only (EUROSCORE I and II, STS, ACEF, and ACEF II) [4,5,6,7,8,9,10,11], thus excluding from the prediction model the effects of surgery on patient outcomes. On the contrary, Lamarche et al. (2017) recently demonstrated that the inclusion of intra-operatory variables improves predictive performance of perioperative models [12].

We propose to follow the evolution of patients clinical status over time, developing two prediction models for the probability of in-hospital death in two pivotal moments: before surgery at operatory theatre (OT) admission (pre-operative model) and at discharge from operatory theatre and admission into Intensive Care Units (ICUs) (post-operative model). Accordingly, the difference in predicted mortality between the two models could be referred as a proxy of the effect of the surgical act on patient outcomes. 

The objective of this study is to propose the use of these two models as a tool to benchmark the performance of the surgical team among hospitals, and identify possible pitfalls in one of the critical aspects of a complex and expensive perioperative care pathway.

## 2. Methods

### 2.1. Ethical Statement

The Margherita-Prosafe Project was approved by the Ethical Committee of the coordinating centre, Comitato Etico Regione Liguria approval n. 381REG2015 on 17 September 2015.

### 2.2. Inclusion and Exclusion Criteria

All patients aged over 16 years admitted to cardiosurgical ICUs joining the Italian Group for the Evaluation of Interventions in Intensive Care medicine (GiViTI) in 2016 or 2017 after cardiac surgery were considered eligible for the analysis. In the case of re-admissions, only the first admission to the ICU was considered. We excluded patients undergoing surgery or endovascular aortic repair for Type-B aortic dissection and patients admitted to the ICU before cardiac surgery.

### 2.3. Data Collection

Clinical information was collected by means of a software (PROSAFE) developed by the GiViTI Coordination Centre. We considered demographics, comorbidities, clinical conditions and organ failures at ICU admission, relevant details concerning cardiac surgery procedures, and hospital mortality. Additional information, including type of procedures, complications during ICU stay, and ICU mortality, was also collected through PROSAFE, but not included in the prognostic models because we aimed to adjust for patients’ features before surgical procedures and at ICU admission.

### 2.4. Data Validity

Data validity was assessed at different stages to avoid selection biases and input error and to guarantee the internal consistency of the records. We excluded all patients admitted within months where more than 10% of admitted patients had incomplete records.

### 2.5. Outcomes

The main outcome was the difference between expected in-hospital deaths computed after and before surgical procedure, for each ICU participating in the project. Differences in mortality were also investigated in subgroups of patients: elective and non-elective surgery and type of surgical procedure: plastic/replacement of aortic valve (AVR), plastic/replacement of mitral valve (MVP and MVR), coronary artery bypass graft (CABG), and ascending aortic surgery.

### 2.6. Statistical Analysis

Categorical variables are reported as frequency and percentage, continuous variables as median and interquartile range (IQR), as appropriate. Variable distributions between alive and dead patients were compared using Wilcoxon–Mann–Whitney test for continuous variables and the chi-squared test for categorical variables.

We estimated the probability of hospital death before and after the cardiac surgical procedure using two logistic regression models. The dataset was randomly split in training and validation sets, containing 85% and 15% of the records, respectively. To develop the former model, we tested only variables available before surgery (e.g., demographics, comorbidities, and type of cardiac surgery). Variables representing rare events (less than 100 patients in the corresponding subgroup or less than 30 events per subgroup) were excluded. The association of each variable with outcome was assessed through bi-variate logistic regression and variables with *p*-values greater than 0.25 were discarded. We tested the linearity of logit as a function of continuous variables, replacing non-linear relationships with piece-wise linear functions. Forward and backward stepwise selection was adopted to identify variables significantly associated with outcome (*p* < 0.01). The levels of categorical variables were merged on the basis of clinical reasoning if their odds ratios were not statistically different. To ease the clinical interpretation of the models in the post-operative model, we included all the variables selected in the pre-operative model, regardless of their statistical significance. The lists of variables tested in the forward and backward selection for both models is reported in Supplementary Material File S1.

The calibration of the model was tested overall and in subgroups of patients, as defined by included variables and by clinical relevance, using the GiViTI calibration belt and test [13,14]. Discrimination was investigated by measuring the Area Under the Curve (AUC) in the Receiver Operator Characteristics (ROC) analysis. Calibration and discrimination was assessed both on the training and in the validation set.

Using the expected probabilities computed with the two models, we evaluated for each ICU and for each subgroup of patients the expected number of death pre-surgery (*e*_pre_ and *e*_post_) and post-surgery. The relative difference in expected mortality was computed as *d* = (*e*_post_ − *e*_pre_)/*e*_pre_ to the 95% confidence interval of *d* was estimated by bootstrap analysis, constructing 1000 pre-surgery and 1000 post-surgery models on 1000 simulated datasets, each one with *N* records randomly resampled from the original dataset with replacement.

## 3. Results

### 3.1. Study Population

**Figure 1:**
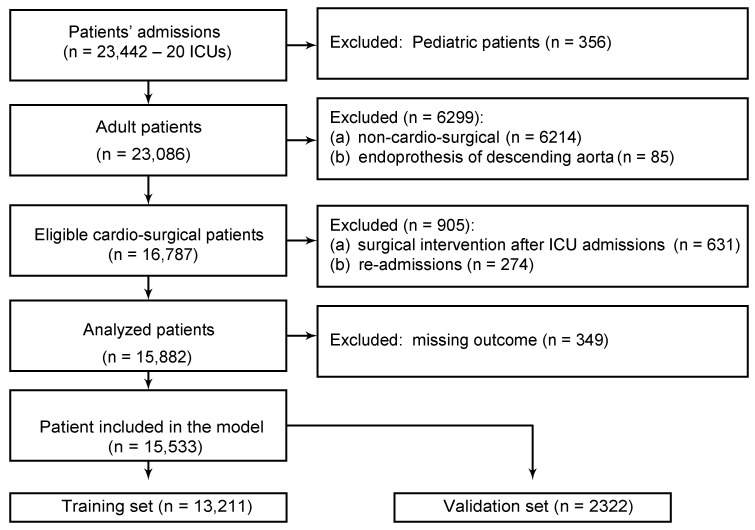
Study flowchart.

Between 2016–2017, we collected data from 23,086 adult patients from 20 centers. Among them, 16,787 patients underwent a cardio-surgical intervention different from the endoprothesis of descending aorta. According to exclusion criteria listed in the Methods section, we excluded patients admitted to the ICU before the interventions and readmissions; thus, we analyzed 15,882 patients (Figure 1). The models were developed on 15,533 patients with non-missing outcomes, using 13,211 records for training and 2322 for validation.

### 3.2. Patients’ Characteristics and Prognostic Models

**Figure 2:**
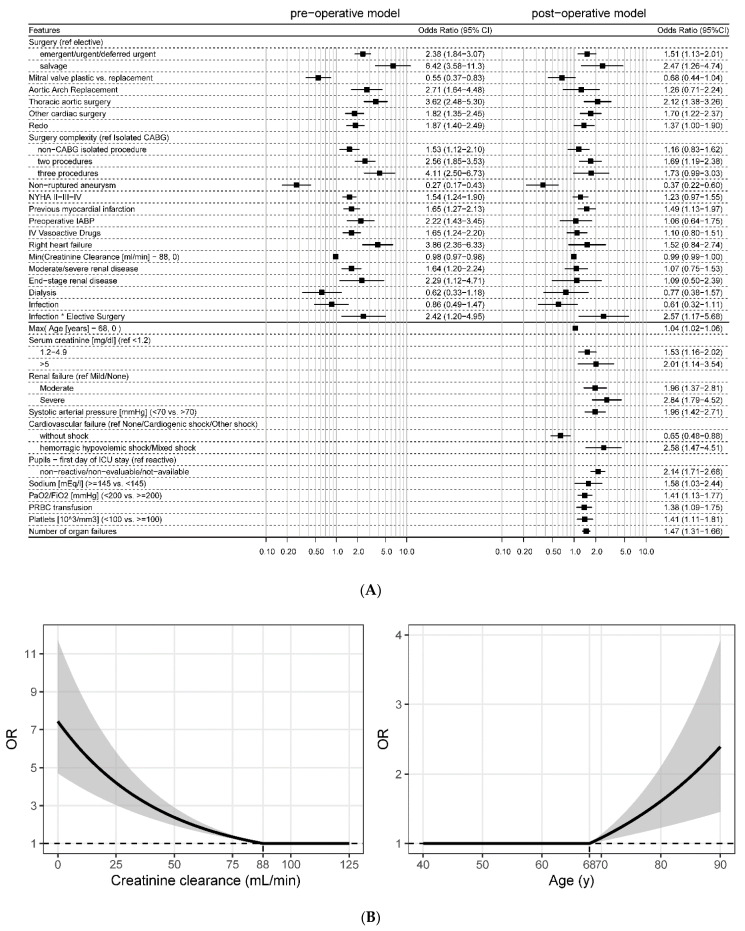
Odds ratios of logistic regression models to predict in-hospital mortality for cardio-surgical patients before surgical act and at ICU admission. (**A**) Forest plot summarizing ORs of multivariate pre-operative (left) and post-operative model (right); (**B**) ORs of continuous variables: creatinine clearance (left) and age (right).

Patients’ demographics, preoperative characteristics, and outcomes are described in Table 1. Figure 2A reports pre-operative (left) and post-operative variables (right) significantly associated with outcome. The odds ratios (OR) of continuous variables, creatinine clearance, and age is plotted in Figure 2B. Patients’ features included in the model and not present in Table 1 are described in Supplementary Material File S2. The AUC is 0.81 and 0.87 on the training set for the pre- and post-operative models, respectively. The *p*-value of the GiViTI calibration test is 0.17 and 0.74 on the training set for the pre- and post-operative models, respectively. On the validation set, the AUC is 0.79 and 0.84 and the *p*-value of the calibration test is 0.07 and 0.10, respectively. ROC curves and GiViTI calibration belts are reported in Supplementary Material File S3.

### 3.3. Difference between Pre- and Post-Operative Mortality

**Figure 3:**
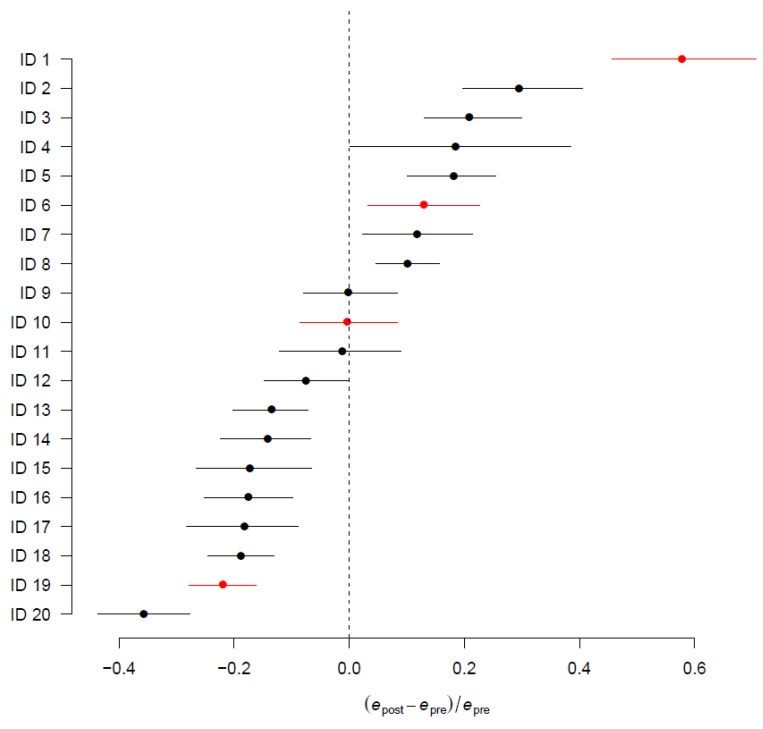
Forest plot summarizing the intraoperative performance *d* = (*e*_post_ − *e*_pre_)/*e*_pre_ for each center, measuring the difference d in expected mortality between pre- and post-operative models at OTR discharge admission and OTR admission discharge (*e*_post_ and *e*_pre_, respectively), normalized by ORT expected pre-operative mortality epre. In red, we highlighted the four centers reported as examples in Figure 4.

The difference in pre-operative and post-operative expected mortality for each center, normalized by pre-operative expected mortality, is reported in Figure 3. The difference between these two predictive models is a proxy of the intraoperative performance for each center.

Centers with lower post-operative mortality are in the left region of the plot, while centers with increased mortality after surgery are toward the right of the plot. Centers 9, 10, and 11 demonstrate similar pre- and post-operative mortality.

### 3.4. Subgroup and Centre-Specific Analysis

Further analyses were performed to identify possible sub-groups of patients influencing perioperative mortality within each center.

**Figure 4:**
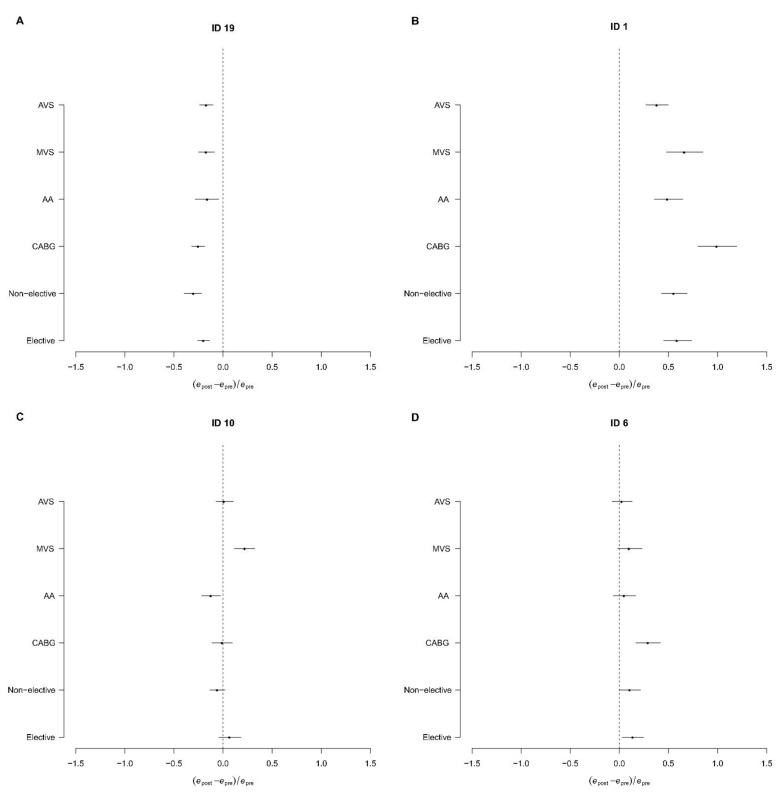
Intraoperative performance d = (*e*_post_ − *e*_pre_)/*e*_pre_. Application of the double-fold model (see Methods section and caption of Figure 3). (**A**) In center 19, surgical activity reduces mortality in all groups; (**B**) in center 1, surgical activity increases mortality in all groups; (**C**) mixed effects on mortality in center 10: MVS was responsible for the overall increase in mortality; (**D**) increased risk of deaths only in CABG patients in center 6. AVS: aortic valve surgery; MVS: mitral valve surgery; AA = ascending aorta; CABG: Coronary Artery Bypass Grafting.

For practical reasons, we present data from four centers (center identifiers 1–6–10–19) to illustrate the findings of our double-fold pre- and post-operative model. Figure 4A shows the subgroup analysis for center 19, where the patient probability of death is consistently reduced after surgery in the overall population and for different subgroups of patients. Figure 4B reports the performance of center 1, where the probability of death increases after the surgical act in all subgroups of patients.

Center 10 has similar pre- and post-operative mortality when considering the overall model (Figure 3). However, when considering sub-analyses according to types of intervention, mitral valve surgery (MVS) increases post-operative mortality compared to pre-operative prediction (Figure 4C). When analyzing different types of intervention (MVP vs. MVR) separately, the effect on mortality was related with MVR only, as MVP patients reach good postoperative outcomes. Indeed, MVR had 0.34 relative mortality reduction between pre- and post-operative models (95% CI 0.22–0.41, 133 patients; 12.6 and 16.7 expected deaths with the pre- and post-operative models, respectively). MVP had −0.13 relative difference in mortality between pre- and post-operative models (95% CI −0.28, −0.015, 131 patients; 4.2 and 3.7 expected deaths in the pre- and post-operative models, respectively).

Center 10 is characterized by a prominent surgeon expert in mitral valve procedures. When calculating mortality according to procedure and to lead surgeon, the most experienced surgeon reduced mortality in MVP, while slightly increasing mortality in MVR cases. Indeed, the pre- and post-operative differences for MVR mortality were 0.25 for this surgeon (95% CI: 0.13, 0.38; 44 patients, 5.7 pre-operative expected deaths and 7.1 post-operative expected deaths) and 0.41 for all other surgeons combined (95% CI: 0.28, 0.53; 89 patients, 7.0 pre-operative expected deaths and 9.8 post-operative expected deaths).

For MVP, the pre- and post-operative difference was −0.21 (95% CI: −0.38, −0.06; 93 patients, 2.6 pre-operative expected deaths and 2.1 post-operative expected deaths) for this surgeon and 0.01 (95% CI: −0.27, 0.35; 38 patients, 1.5 pre-operative expected deaths and 1.6 post-operative expected deaths) for all other surgeons combined.

Center 6 reached a lower overall performance compared to the mean performance of the centers, with an increase in odds ratio of 0.13 (95% CI: 0.03, 0.27; 1027 patients, 51.4 pre-operative expected deaths and 58.1 post-operative expected deaths). Sub-analyses reported in Fig. 4d shows significantly increased risk of death in CABG patients only (difference 0.29, 95% CI: 0.17, 0.42; 354 patients, 15.2 pre-operative expected deaths and 19.6 post-operative expected deaths) and in elective patients (difference 0.14, 95% CI: 0.03, 0.25; 909 patients, 35.4 pre-operative expected deaths and 40.3 post-operative expected deaths), with elective CABG reporting a 0.31 difference in the odds ratio (95% CI: 0.18, 0.45; 312 patients, 11.5 pre-operative expected deaths and 15.1 post-operative expected deaths).

No significant increase in mortality was detected in elective non-CABG patients (difference 0.06, 95% CI: −0.05, −0.16; 597 patients, 23.9 pre-operative expected deaths and 25.3 post-operative expected deaths).

## 4. Discussion

Our results show that the predicted post-operative mortality differs from the predicted pre-operative mortality. The distance between the two probabilities expresses the impact of surgery on patient outcomes, and may be used to benchmark intra-operative performances.

Surgery is the sum of both organizational factors (hospital services including blood bank and ancillary services for operatory block) and competences from several professionals, such as cardiac surgeons, cardiac anesthesiologist, nurses, perfusionists, and healthcare assistants. These aspects are closely linked to the level of resources, and while it is difficult to unbundle the role of each team component, it is clear that the first surgical operator has a major role in overall surgical performance [15,16].

The new tool proposed in this work allows to identify significant differences between centers from our cohort either in the overall population or in subgroup analysis, as exemplified in Figure 3.

When the (either good or bad) performance of a centre is consistent in all patient groups, one may argue that protective or detrimental factors are spread throughout the whole care pathway.

Subgroup analyses may detect specific areas of excellence or critical issues in centers where the overall mortality may appear within a range of normality.

This is the case of center 10 (Figure 4C), where our models were able to identify differences in the performance when stratifying by either the type of intervention or by the surgical team. Specifically, patients undergoing a certain surgical intervention had a lower mortality when treated by a team including a surgeon experienced in that cardiac surgical technique.

This example suggests, as expected, that it is reasonable to attribute to the lead surgeon and to his/her team a significant portion of the responsibility of overall intraoperative performance, and that the development of ultra-specialistic technique may represent both an intellectual investment and a productive resource, first and foremost in health terms, for the medical service.

### Limitations

Intraoperative deaths were excluded from the analysis, as they lack post-operative data. However, intraoperative mortality in our population was low, in accordance with the published literature for elective, urgent and emergent cardiac surgery cases.

In our model, we considered only data available at admission and discharge from the operatory theatre. Other studies considered pre-hospital variables, including schooling, economic status and social and family status (being single, socially isolated or without caregiver), which may have a weight in prognostic scores [17,18].

Moreover, we acknowledge that our system for data collection represents a significant workload for each center, given the large amount of collected information and the number of internal quality checks. On the one hand, this may hinder participation, while on the other, it ensures data quality.

## 5. Conclusions

We built a double-fold predictive tool that is useful to evaluate hospital performance at different moments of the cardiac surgery perioperative pathway, thus allowing to analyze the contribution of the intraoperative performance. Our model allows different subgroups of patients and distinct types of surgery to be analyzed, promoting improvement through the recognition of possible quality barriers and through the identification and reinforcement of good and exportable clinical practices.

## Figures and Tables

**Table 1:** Patient description and comparison between alive and dead patients (*p*-values were computed with Wilcoxon–Mann–Whitney test for the continuous variables and chi-squared test for the categorical variables.

	Total (N = 15,533)	Alive (N = 14,971)	Dead (N = 562)	*p*-Value
Age				<0.001
Median (Q1, Q3)	70 (62, 76)	70 (62, 76)	73 (65, 78)	
Gender (Male)	10,506 (67.6%)	10,143 (67.8%)	363 (64.6%)	0.116
BMI				0.141
Underweight	522 (3.4%)	506 (3.4%)	16 (2.9%)	
Normal	6617 (42.8%)	6351 (42.7%)	266 (47.6%)	
Overweight	5809 (37.6%)	5614 (37.7%)	195 (34.9%)	
Obese	2498 (16.2%)	2416 (16.2%)	82 (14.7%)	
Missing	87	84	3	
Hypertension	11,364 (73.2%)	10,951 (73.1%)	413 (73.5%)	0.858
NYHA class				<0.001
I	7798 (50.2%)	7600 (50.8%)	198 (35.2%)	
II–III	7198 (46.3%)	6919 (46.2%)	279 (49.6%)	
IV	537 (3.5%)	452 (3.0%)	85 (15.1%)	
Previous myocardial infarction	2577 (16.6%)	2452 (16.4%)	125 (22.2%)	<0.001
Arrhythmia	2376 (15.3%)	2238 (14.9%)	138 (24.6%)	<0.001
Diabetes				0.006
None	12,076 (77.7%)	11,656 (77.9%)	420 (74.7%)	
Type 1	132 (0.8%)	124 (0.8%)	8 (1.4%)	
Type 2 without insulin treatment	2258 (14.5%)	2181 (14.6%)	77 (13.7%)	
Type 2 with insulin treatment	1067 (6.9%)	1010 (6.7%)	57 (10.1%)	
Ejection fraction				<0.001
<30%	397 (2.6%)	357 (2.4%)	40 (7.1%)	
30–50%	4611 (29.7%)	4383 (29.3%)	228 (40.6%)	
>50%	10,525 (67.8%)	10,231 (68.3%)	294 (52.3%)	
Serum creatinine (mg/dL)				<0.001
Median (Q1, Q3)	1 (1, 1)	1 (1, 1)	1 (1, 2)	
Missing	4	4	0	
Creatinine clearance (mL/min) (computed with Cokcroft–Gault formula)				<0.001
Median (Q1, Q3)	74 (55, 95)	74 (56, 95)	56 (39, 76)	
Missing	4	4	0	
Urgency of intervention				<0.001
Elective	13,021 (83.8%)	12,696 (84.8%)	325 (57.8%)	
Deferred urgent	1329 (8.6%)	1252 (8.4%)	77 (13.7%)	
Emergent/urgent	1083 (7.0%)	960 (6.4%)	123 (21.9%)	
Salvage	100 (0.6%)	63 (0.4%)	37 (6.6%)	
Redo	1064 (6.8%)	974 (6.5%)	90 (16.0%)	<0.001
Valve surgery	8551 (55.1%)	8252 (55.1%)	299 (53.2%)	0.370
Aortic repair	206 (1.3%)	199 (1.3%)	7 (1.2%)	0.865
Aortic replacement	5468 (35.2%)	5264 (35.2%)	204 (36.3%)	0.579
Mitral repair	1732 (11.2%)	1698 (11.3%)	34 (6.0%)	<0.001
Mitral replacement	1809 (11.6%)	1705 (11.4%)	104 (18.5%)	<0.001
Tricuspid repair	504 (3.2%)	471 (3.1%)	33 (5.9%)	<0.001
Tricuspid replacement	34 (0.2%)	29 (0.2%)	5 (0.9%)	<0.001
CABG	7454 (48.0%)	7227 (48.3%)	227 (40.4%)	<0.001
Thoracic aorta surgery	1748 (11.3%)	1617 (10.8%)	131 (23.3%)	<0.001
Other cardiac surgery	1023 (6.6%)	927 (6.2%)	96 (17.1%)	<0.001
length of ICU stay				<0.001
Median (Q1, Q3)	1 (1, 2)	1 (1, 2)	4 (1, 12)	
Hospital length of stay (copy)				<0.001
Median (Q1, Q3)	11 (8, 17)	11 (8, 17)	16 (7, 32)	
Missing	7	0	7	
ICU outcome	315 (2.0%)	0 (0.0%)	315 (56.0%)	<0.001
Hospital outcome	562 (3.6%)	0 (0.0%)	562 (100.0%)	<0.001

BMI = Body Mass Index, CABG = Coronary Artery Bypass Graft, NYHA = New York Heart Association, ICU = Intensive Care Unit.

## Data Availability

Data availability upon reasonable request. Data cannot be downloaded but only accessed within IRCCS Mario Negri network.

## Supplementary Materials

The following supporting information can be downloaded at: https://www.mdpi.com/article/10.3390/jcm11113231/s1, Supplementary Material File S1: Variables tested in the forward/backward selection in both pre-operative and post-operative models. Supplementary Material File S2: Patients’ features included in the model and not present in manuscript Table 1. Supplementary Material File S3: Discrimination and calibration analysis for training and validation datasets.

## Appendix A

Cardiac Surgical Intensive Care Writing Committee (GiViTI): Giuseppe Buscaglia, IRCCS Azienda Ospedaliera Universitaria San Martino Ist., Genova; Fabio Mencarelli, Azienda Ospedaliera di Perugia, Perugia; Gian Paolo Castelli, ASST Mantova, Mantova; Sandra Nonini, Niguarda Ca’ Granda, Milano; Paolo Del Sarto, G. Pasquinucci Heart Hospital FTGM, Massa; Paolo Prati, Policlinico Tor Vergata, Roma; Marco Maurelli, Fondazione IRCCS Policlinico San Matteo, Pavia; Paolo Pessano, APSS-Ospedale S. Chiara, Trento; Antonio Arcadipane, ISMETT, Palermo; Andrea Balata, Ospedale Civili SS. Annunziata-ASL 1, Sassari; Ricardo Martinez Escobar, Fondazione Poliambulanza Istituto Ospedaliero, Brescia; Arianna Abascià, Ospedale Mauriziano Umberto I, Torino; Anna Zamperoni, Ospedale Regionale Caà Foncello, Treviso; Gianluigi Redaelli, San Gerardo dei Tintori, Monza; Roberto Agostinelli, Istituto Clinico San Rocco, Ome; Alessandro Rech, A.O. Ospedale di Circolo Fondazione Macchi, Varese; Claudio Fiorelli, Azienda Ospedaliera Santa Maria, Terni; Carolina Monaco, Azienda Ospedaliero Universitaria Maggiore, Novara; Andrea Vardanega, Ospedale dell’Angelo, Venezia; Matteo Lucchelli, Azienda Ospedaliera-Ospedale Civile di Legnano, Legnano.
