# Supplementary material for: Benchmark of Intraoperative Activity in Cardiac Surgery: A Comparison between Pre- and Post-Operative Prognostic Models
Source: J Clin Med. 2022 Jun 6;11(11):3231. doi: 10.3390/jcm11113231 (PMC9181738; doi:10.3390/jcm11113231)
Supplement: Supplementary file 1 [file jcm-11-03231-s001.zip › jcm-1706326-supplementary.pdf]

SM1: Supplemental Material S1
Variables tested in the forward/backward selection in both pre-operative and post-operative models

Demographics
Age
Sex

Comorbidities
Diabetes
COPD
Moderate/severe renal disease
End-stage renal disease
NYHA II-III-IV
Previous myocardial infarction
Arrhythmia
Cerebrovascular disease
Drug-induced coagulopathy
Peripheral vascular disease

Clinical conditions
Asymptomatic Coronary Artery Disease
Non-traumatic pericardial effusion
Pulmonary hypertension
Right heart failure
Left heart failure
Acute ischemia
Acute myocardial infarction
Coronary artery disease
Aortic pathology
Aortic valve insufficiency
Aortic valve stenosis
Mitral valve insufficiency
Mitral valve stenosis
Tricuspid valve insufficiency
Left coronary artery disease
Non-ruptured aneurysm
Ruptured aneurysm
Infections
Endocarditis

Type of surgery
Aortic Arch Replacement
Ascending aorta replacement
CABG
Mitral valve plastic
Mitral valve replacement
Tricuspid valve plastic

Tricuspid valve replacement
Thoracic aortic surgery
Other cardiac surgery
Surgery complexity (Isolated CABG, non-CABG isolated procedure, two procedures, three procedures)
Timing of surgery (elective, emergent, urgent, deferred, urgent, salvage)
Redo

Other variables
Body Mass Index
Body Surface Area
Pre-operative hospital length of stay
Dialysis
Renal damage
Creatinine Clearance
Serum creatinine
Ejection fraction
IV Vasoactive Drugs
Anticoagulant therapy
Antiplatelet therapy
Preoperative IABP
Critical pre-operative conditions
Number of pathological coronary arteries

Variables tested in the forward/backward selection only in the post-operative model

Intra-operative devices/treatment/variables
PRBC transfusion (yes/no, amount)
FFP transfusion (yes/no, amount)
Platelet transfusion (yes/no, amount)
Fibrinogen transfusion (yes/no, amount)
BCFC transfusion (yes/no, amount)
Transesophageal echocardiography
Platelet function test
Thromboelastometry/Thromboelastography
Aortic cross-clamp (yes/no, length)
Length of ECC
Deep hypothermic circulatory arrest (yes/no, length)
Cerebral perfusion
Serum lactate
Bleeding
Sternal diastasis at ICU admission

Clinical parameters in the first 24 hours of ICU stay
Systolic blood pressure
Mean arterial pressure

Serum creatinine
Platelets
PaO2/FiO2
Heart rate
Serum potassium
Serum sodium
Serum HCO3
Serum bilirubin
Serum creatinine
Urine output
White blood cell count
Platelet count
Blood urea nitrogen
Pupil reactivity - first day of ICU stay

Organ failures
Number of organ failures
Renal failure (mild, moderate, severe)
Cardiovascular failure (none, without shock, cardiogenic shock, hemorrhagic shock)
Respiratory failure (none, hypoxic, hypercapnic)
Metabolic failure
Neurological failure

Other variables
Source ward
Intervention time
Duration of intervention

Supplemental material S2: Other patients' features included in the model and not present in manuscript Table 1

	Total (N=15533)	Alive (N=14971)	Dead (N=562)	p value
**Renal disease**				< 0.001
None	14505 (93.4%)	14055 (93.9%)	450 (80.1%)	
Moderate/severe	874 (5.6%)	791 (5.3%)	83 (14.8%)	
End-stage	154 (1.0%)	125 (0.8%)	29 (5.2%)	
**Non-ruptured aneurysm**	1433 (9.2%)	1386 (9.3%)	47 (8.4%)	0.472
**Right heart failure**	159 (1.0%)	125 (0.8%)	34 (6.0%)	< 0.001
**Infection**	544 (3.5%)	493 (3.3%)	51 (9.1%)	< 0.001
**Surgery complexity**				< 0.001
Isolated CABG	5233 (33.7%)	5128 (34.3%)	105 (18.7%)	
non-CABG isolated procedure	6359 (40.9%)	6120 (40.9%)	239 (42.5%)	
two procedures	3461 (22.3%)	3282 (21.9%)	179 (31.9%)	
three procedures	480 (3.1%)	441 (2.9%)	39 (6.9%)	
**IV Vasoactive Drugs**	1148 (7.4%)	1056 (7.1%)	92 (16.4%)	< 0.001
**Dialysis**	277 (1.8%)	239 (1.6%)	38 (6.8%)	< 0.001
**Preoperative IABP**	315 (2.0%)	273 (1.8%)	42 (7.5%)	< 0.001
**Systolic arterial pressure (< 70 mmHg)**	521 (3.4%)	393 (2.6%)	128 (22.8%)	< 0.001
**PaO2/FiO2 [mmHg] (< 200)**	3559 (22.9%)	3312 (22.1%)	247 (44.0%)	< 0.001
**Serum creatinine [mg/dl]**				< 0.001
< 1.2	11755 (75.7%)	11581 (77.4%)	174 (31.0%)	
1.2 - 4.9	3444 (22.2%)	3164 (21.1%)	280 (49.8%)	
> 5	334 (2.2%)	226 (1.5%)	108 (19.2%)	
**Platelets (< 100x10^3/mm3)**	2122 (13.7%)	1910 (12.8%)	212 (37.7%)	< 0.001
**Sodium (>=145 mEq/l)**	399 (2.6%)	342 (2.3%)	57 (10.1%)	< 0.001
**Pupils - first day of ICU stay (non-reactive/non-evaluable/not-available)**	2968 (19.1%)	2677 (17.9%)	291 (51.8%)	< 0.001
**Renal failure**				< 0.001
Mild/None	14675 (94.5%)	14344 (95.8%)	331 (58.9%)	
Moderate	439 (2.8%)	348 (2.3%)	91 (16.2%)	
Severe	419 (2.7%)	279 (1.9%)	140 (24.9%)	
**Cardiovascular failure**				< 0.001

Mild/None	13233 (85.2%)	12836 (85.7%)	397 (70.6%)	
Moderate	2185 (14.1%)	2070 (13.8%)	115 (20.5%)	
Severe	115 (0.7%)	65 (0.4%)	50 (8.9%)	
**Number of organ failures**				< 0.001
Median (Q1, Q3)	0 (0, 1)	0 (0, 1)	2 (1, 4)	
**PRBC transfusion**				< 0.001
No	10426 (67.3%)	10227 (68.5%)	199 (35.5%)	
Yes	5068 (32.7%)	4707 (31.5%)	361 (64.5%)	
Missing	39	37	2	
**Hospital outcome**	562 (3.6%)	0 (0.0%)	562 (100.0%)	< 0.001

Supplemental Material S3: Discrimination and calibration analysis for training and validation datasets

Training set

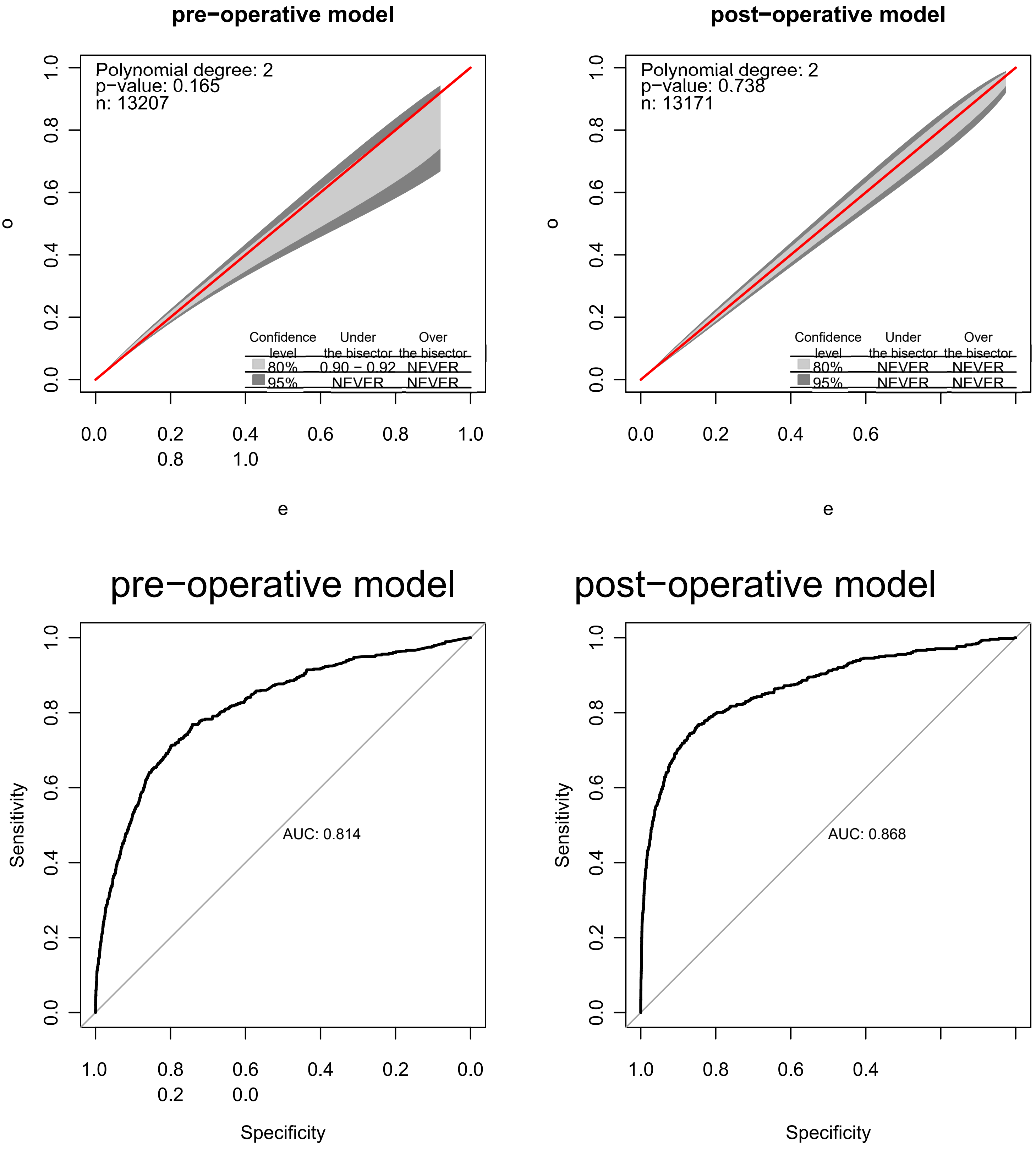

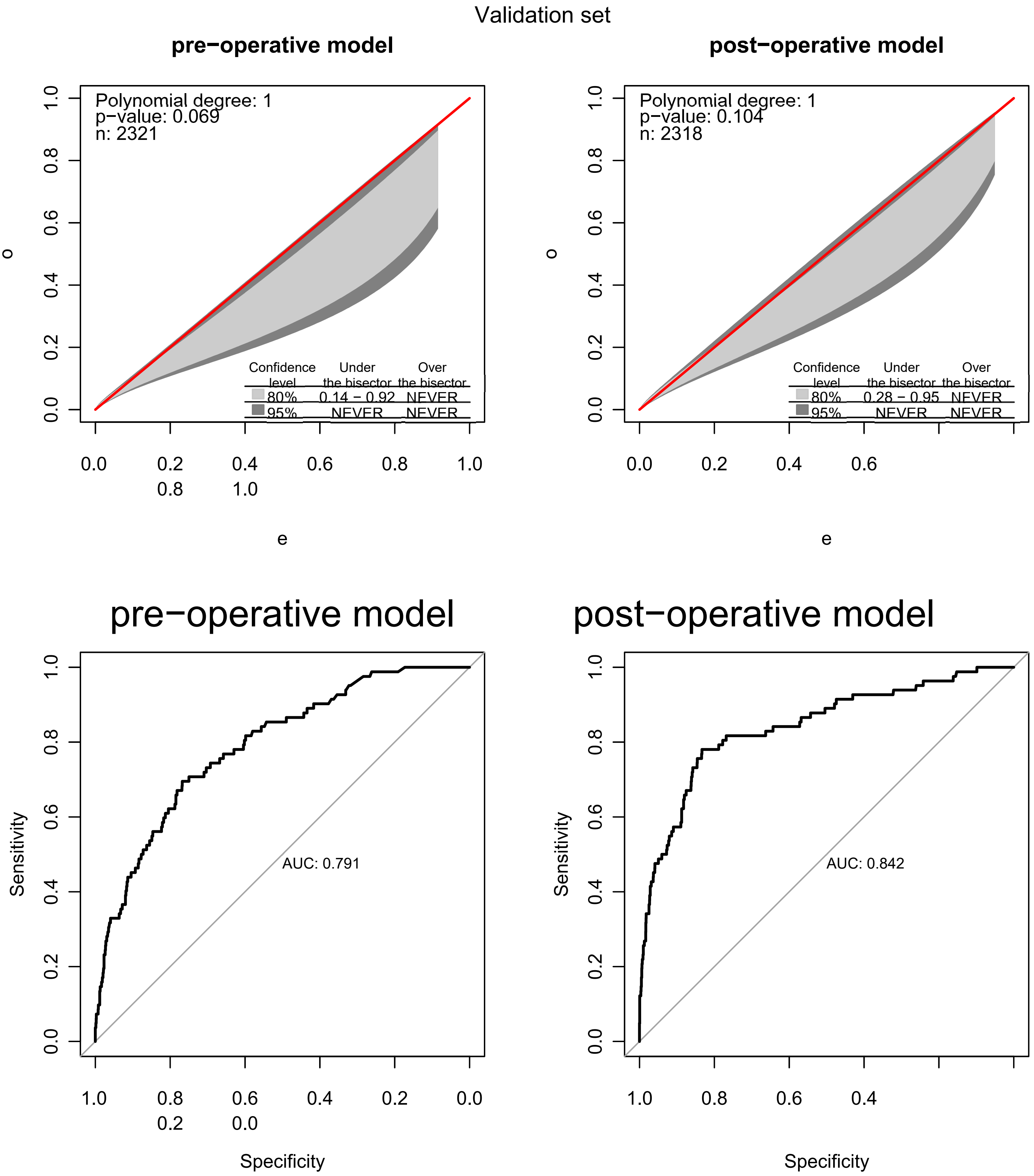
Validation set
pre−operative model
Polynomial degree: 1
p−value: 0.069
n: 2321
Confidence level
Under the bisector
Over the bisector
80%
0.14 − 0.92
NEVER
95%
NEVER
NEVER
o
e
post−operative model
Polynomial degree: 1
p−value: 0.104
n: 2318
Confidence level
Under the bisector
Over the bisector
80%
0.28 − 0.95
NEVER
95%
NEVER
NEVER
o
e
pre−operative model
AUC: 0.791
Sensitivity
Specificity
post−operative model
AUC: 0.842
Sensitivity
Specificity
